# One out of every three adult TB patients suffered from undernutrition in conflict affected Southern Ethiopia: a multicenter facility-based cross-sectional study

**DOI:** 10.3389/fepid.2025.1405845

**Published:** 2025-06-13

**Authors:** Awoke Abraham, Tagese Yakob, Desalegn Dawit, Adisu Ashiko, Daniel Tekese, Eskinder Israell

**Affiliations:** ^1^ Division of Nutrition, Maternal and Child Health Unit, Wolaita Zone Health Department, Wolaita Sodo, Ethiopia; ^2^ SCOPE Registrar, World Food Programme (WFP), Addis Ababa, Ethiopia; ^3^ School of Public Health, College of Health Science and Medicine, Hawassa University, Hawassa, Ethiopia; ^4^ Public Sector Strengthening, Marie-stopes International (MSI), Hawassa, Ethiopia; ^5^ School of Public Health, College of Health Science and Medicine, Wolaita Sodo University, Wolaita Sodo, Ethiopia

**Keywords:** undernutrition, conflict, tuberculosis, associated factors, Ethiopia

## Abstract

**Background:**

Although tuberculosis mortality has dramatically decreased over the last decade, tuberculosis remains the world's biggest cause of death. Conflict affected nations hold vast majority of malnourished people globally, where many people die each year of tuberculosis. With regard to the global burden of tuberculosis, Ethiopia ranks third in the African continent and seventh overall. But in the research arena, the severity of the issue is not as well understood. Therefore, the current study aimed to assess undernutrition and the determinant factors among adult TB-patients receiving treatment in public health facilities in conflict affected zones of Southern.

**Methods:**

A multicenter facility-based cross-sectional study was conducted from 27/08/2023–28/ 09/2023 among 414 randomly selected adult (age ≥18 years) TB-patients receiving treatment at public health facilities in conflict affected zones of Southern Ethiopia. An interviewer-administered questionnaire and anthropometric measurements were used to collect data from study participants after written informed consent provision. By using SPSS Version 25, bivariate and multivariable logistic regression models were employed to determine the factors related to nutritional status.

**Results:**

Overall, 33.3% of study participants had undernutrition, with a [95% CI (28.8%–38.1%)]. Factors such as cigarette smoking [AOR = 2.02, 95% CI; 1.22, 3.34] chat chewing [AOR = 2.50, 95% CI; 1.59, 3.93] regular cheka drinking [AOR = 1.82; 95% CI, 1.22–2.71] and household food insecurity [AOR = 1.78, 95% CI; 1.19, 2.66] had significant association with undernutrition.

**Conclusions:**

The results of this study show that undernutrition affects one in three adult TB patients. Lifestyle factors such as smoking and chewing, and dietary factors like cheka eating and household food security had significant association with undernutrition. In order to improve the quality of life for TB patients, it is imperative that all stakeholders should prioritize addressing the lifestyle and nutritional aspects that are essential to the effectiveness of TB control and prevention initiatives.

## Background

Undernutrition is the state in which an individual's intake of nutrients and energy is insufficient to meet their demands in order to maintain good health (1). Approximately 390 million persons over the age of 18 were underweight globally in 2022. An additional 190 million had thinness (BMI-for-age more than two standard deviations below the reference median) (2).

Worldwide, TB has probably returned to being the world's leading cause of death from a single infectious agent, following three years in which it was replaced by coronavirus disease (COVID-19) (3). Being a major cause of premature adult population death and affecting individuals worldwide, it is a huge public health concern (4). In the last two years, about 10 million people worldwide have been diagnosed with tuberculosis, and 1.4 million have died from the disease (5). Nonetheless, the majority of the disease burden is found in developing nations, where over 50% of cases occur in economically productive age ranges (6). With regard to the global burden of tuberculosis, Ethiopia ranks third in the African continent and seventh overall (7). Remarkably, tuberculosis (TB) ranks third in hospital fatalities and is the eighth most common cause of hospital admissions in Ethiopia (8).

Undernutrition and tuberculosis (TB) are interconnected, with undernutrition increasing TB risk and malnutrition resulting from the latter (9, 10). A sufficient, well-balanced diet has been associated with faster sputum conversion and improved weight gain (11). Supplements high in energy also improved athletic performance and lean body mass (12). In addition, TB patients who do not receive adequate energy have been found to regularly have poor adherence to anti-TB medicine. This may raise the patient's risk of developing multi-drug resistant (MDR) type tuberculosis (TB), which is presently one of the major public health problems challenging Ethiopia and the rest of the world (13, 14).

Globally, armed conflicts claimed the lives of almost 191 million individuals during the 20th century (15). Armed conflicts degrade housing, sanitation, and hygiene, interrupt prevention programs, demolish infrastructure, and destroy existing healthcare systems, all of which contribute to the spread of infectious illnesses like tuberculosis (16). Furthermore, during armed conflicts, the burden of tuberculosis increases due to overcrowding and malnutrition (17). Despite Ethiopia being the oldest sovereign state on the African continent, conflict has become the norm (18). It wouldn't be an overstatement to suggest that war and conflict have characterized Ethiopia's history (19). Currently, in addition to on-going internal conflict, neighbour Sudanese conflict and economic slowdowns as a result of trade wars, the Russian—Ukraine war (20, 21), and a global pandemic like COVID-19 (22, 23) raised the rate of related hunger. In conflict affected areas, TB is also one of the most well-known causes of illness and mortality (24–26).

Undernutrition was more common among tuberculosis patients who were male, from low-income families, had low educational attainment, were unable to work, and did not receive nutritional counseling (27–29). Behavioral characteristics were also linked to undernutrition. For example, smoking increases the likelihood of consuming insufficient calories, which has been connected to a decrease in appetite and an increase in resting energy expenditure due to nicotine's effects on the body's metabolism (30, 31).

For tuberculosis patients to have a lower chance of comorbidities and related mortality as well as negative treatment outcomes, their nutritional state must be improved. As undernutrition is powerful predictor of active TB, its reduction among general population can significantly lower the incidence (32). Furthermore, research on the extent and causes of undernutrition is needed to improve early case detection and management; yet, there is a dearth of Ethiopian literature in this area. Eventually, even the previously done researches have a more narrow scope, which diminishes the generalizability of the finding (33, 34).

All tuberculosis patients in Ethiopia are currently eligible for free treatment. Management aspects of tuberculosis control program has to integrate addressing malnutrition, household food insecurity, and other TB determinants and consequences (35).

Developing a suitable intervention to address the worsening effects of adult tuberculosis patients requires magnitude of undernutrition and its associated components. Though undernutrition and tuberculosis are highly prevalent in Ethiopia, relatively little research has been conducted on undernutrition and associated risk factors in adult-TB patients (33, 36). Furthermore, these studies have only looked at other regions, ignoring the conflict-affected Southern regions of the nation, which are experiencing the worst drought the Horn of Africa has seen in decades (37). Therefore, the aim of this study was to assess the detrimental factors associated with undernutrition among adult TB patients receiving treatment at public health facilities in conflict affected zones of Southern Ethiopia, 2023.

## Methodology

### Study area, period and design

From 27th August–28th September 2023 a health facility-based cross-sectional study was carried out in Amaro, Burji, Derashe, Ale and Konso zones, in Southern Ethiopia. These areas were respectively 357 km, 546 km, 550 km and 522 km from south of the country's capital, Addis Ababa.

The research area's population was estimated to be 3,157,673 based on the Central Statistics Agency of Ethiopia's most recent population projection, which included 2,684,076 rural (85%) and 473,597 urban (15%) individuals (38). This area is among zones threatened by drought (39) and, also one of inter-communal conflict-affected Ethiopian zones (40). In this study area, there are 32 health facilities delivering anti-TB services during the study period.

### Eligibility

Source population: All adult (age ≥18 years) TB patients who were receiving treatment in conflict affected zones of Southern Ethiopia during the study period.

Study population: All adult TB patients who were receiving treatment in randomly selected public health facilities.

Adult TB patients who were pregnant and lactating woman, elderly above 60 year old, HIV and diabetic mellitus participant, critically ill and can't tell the necessary information during data collection were excluded from the study.

### Variables of study

#### Dependent variable

Undernutrition [Body Mass Index (BMI) < 18.5] among adult TB patient; BMI was computed by standard procedures i.e., as body weight in kilograms divided by the square of height in meter. Severe undernutrition is defined as a BMI < 16.0 kg/m2, moderate undernutrition as a BMI = 16.0 kg/m^2^–16.99 kg/m^2^, mild undernutrition as a BMI = 17.0 kg/m^2^–18.49 kg/m^2^, Normal weight as a BMI = 18.5 kg/m^2^–24.99 kg/m^2^, Overweight as a BMI = 25.0 kg/m^2^–29.99 kg/m^2^ and Obese as a BMI ≥ 30 kg/m^2^. To make interpretation simpler, we dichotomized the BMI into two categories: undernutrition (BMI < 18.5) and Normal (BMI ≥ 18.5).

#### Independent variables

Socioeconomic and demographic factors included age, sex, education, weight, height, family size and Occupational status.

Dietary practice and Lifestyle characteristics were: Feeding pattern, meal frequency, individual diversity score, regular cheka drinking, and household food security Drinking alcohol, Smoking cigarettes and Chewing chat.

Health history characteristics included Duration on TB treatment, Type of Treatment and Treatment adherence.

### Operational definitions

Tuberculosis patient: who has sign and symptoms of cough, loos of appetite, easily fatigability, night sweating and loos of weight for the last 2 weeks that is verified by radiographic abnormalities compatible with active tuberculosis and smear examination positive for AFB (41).

Chat (Catha edulis) is a recreational, chewed herbal drug that has been used as a psychostimulant for centuries in East Africa and the Arabian Peninsula (42).

Regular chat chewing: For the previous year or longer, khat was chewed at least once every week (43). Cheka is one of the indigenously fermented alcoholic beverages that is extensively consumed and valued by consumers in urban and rural areas of the Konso and neighboring Zones in southern Ethiopia (44).

Meal frequency: This was defined as the number of meals consumed per day. Participants were categorized as having adequate meal frequency if they consumed three or more meals per day and inadequate if they consumed fewer than three meals per day.

Food security: Household food security was assessed using the Household Food Insecurity Access Scale (HFIAS). A household was classified as food secure if the total score was <2 and food insecure if the score was ≥2.

Alcohol use: Participants were classified as alcohol users if they reported consuming alcohol during their TB treatment follow-up.

Adherent: Individuals who received more than 90% of the prescribed dosage (45, 46).

Non- adherent: If patients took less than 90% of the prescribed dosages of their medications, they were considered non-adherent to therapy (45, 46).

### Sample size determination

Our target sample size was 416. This sample size was calculated using a formula for single population proportion by taking a 44.3% proportion (p) of chronic malnutrition; 5% margin of error; 10% non-response rate; and 95% confidence intervals (CI) (47). The same approach was used by similar studies (48, 49). Initially, among 32 healthcare facilities providing TB treatment in four zones during data collection, 11 were randomly selected. Then, a sampling frame was prepared using the patient's medical registration number from the TB registration record of each medical facility. Following that, the total sample size was distributed proportionally to each facility. The study participants were subsequently selected using a computer-generated basic random sampling process from each of the specified healthcare facilities.

### Data collection procedures

A total of 11 research assistants and 04 supervisors assisted with data collection. They were at least diploma holder health professionals who spoke the local language and had previous experience in data collection. Supervisors and data collectors received two days of training at their respective zonal centres regarding the study's goals, the tool's contents, and data collection techniques.

Using a beam balance, the weight of the research subjects was measured to the nearest 0.1 kg while they were not wearing shoes or bulky clothing. Accuracy checks of the weighing scale were also made against a standard weight. Prior to weighing each research subject, calibration was done by setting it to zero. Using a Seca vertical height scale, the study participants' heights were measured while they were standing straight in the centre of the board. Participants were asked to take off their shoes and stand in the Frankfurt plane. A measuring board was touched at the occipital (back of the head), shoulder blades, buttocks, and heels, and the height was recorded to the closest 0.01 cm.

To determine undernutrition, first Body mass index (BMI) of the study subjects was calculated by dividing weight in kilograms by the square of the height in meters (kg/m^2^). Using BMI, undernutrition was defined by BMI < 18.5 kg/m^2^. Moreover, undernutrition was classified: severe (BMI < 16 kg/m^2^), moderate (BMI 16–16.99 kg/m^2^) and mild (BMI 17–18.49 kg/m^2^) (20).

Face-to-face interviews were used to gather Sociodemographic information from patients, and medical records were used to extract clinical features. The Food and Agricultural Organization's recommended 24 h dietary recall method was used to measure each respondent's individual dietary diversity using the standard individual dietary diversity scale items. Adult TB patients were classified as having a low individual dietary variety score if they consumed fewer than seven of the 14 food groups; otherwise, they were not (32).

The standard household food security access scale was utilized in order to evaluate the level of household food security (21). The nine questions on food insecurity are categorized as follows: 0 = no occurrence, 1 = rarely, 2 = often, and 3 = often. A recall span of 30 days, spread across four weeks, was used for each question. The score was then totalled and divided into two groups: households with secure food sources and households with insecure food sources. If the household received a score of two or higher, there was food insecurity (21). All patients received nutritional counselling at the time of the actual data collection. All phases of the data collection were rigorously observed and overseen by the principal investigator and the assigned supervisors.

### Statistical analysis

Statistical Package for Social Sciences (SPSS) Version 25 was used for data analysis. Cross-tabulation and frequencies were used to describe the study population in terms of pertinent factors and to look for missing values in the variables. Additionally, the study population characteristics were summarized using percentages, proportions, and summary statistics. To evaluate the independent relationship between the covariates and the outcome variable, binary logistic regression analysis was performed. To control confounding effects and identify significant factors, variables from the bivariable analysis with *p*-values less than 0.25 were added to the Multivariable Logistic Regression model (22, 23). The Hosmer-Lemeshow test for goodness of fit was used to see whether the model was adequate to fit the outcome variable with the predictors; the result was 0.43, which was within the acceptable range. Statistically significant factors in the multivariable analysis were those with *p*-values less than 0.05, and we reported the corresponding adjusted odds ratios (AOR) along with corresponding 95% confidence intervals (CI).

### Patients and the public involvement

No patients or members of the public were involved in the planning, execution, or distribution of the study's results.

## Results

### Socioeconomic and demographic characteristics of study participants

Out of the 416 potential participants approached, 414 agreed and successfully participated in this study with a response rate of 99.5%. The mean age of the study participants was 31.94 (SD ± 10.97) years, with 18 and 65 years being the minimum and maximum ages, respectively. Of the total (414) respondents two hundred and forty six (59.4%) were men and three hundred twenty eight (79.9%) were living with their spouses. Majority (82.2%) of study participants were living with family size of five or more members (Table 1).

**Table 1:** Socio-demographic characteristics among adult TB patients receiving treatment in public health facilities of conflict affected zones of Southern Ethiopia, 2023 (*n* = 414).

Variable	Characteristics	Frequency(*n* = 414)	Percent
Age	18–35	262	63.3
	>=35	152	36.7
Sex	Male	246	59.4
	Female	168	40.6
Marital Status	Single	74	17.9
	Married	328	79.2
	Divorced	12	2.9
Educational status	Litrate/Primary	202	48.8
	Secondary/Above	212	51.2
Occupational status	Unemployed	104	25.1
	Civil servant	136	32.9
	Daily laborer	88	21.3
	Merchant	86	20.7
Household family size	>=5	357	82.2
	<5	57	13.8

### Dietary and lifestyle characteristics of the participants

Based on their individual dietary diversity score, two hundred and twenty eight (55.1%) of the study participants did not receive a diversified diet. Only one in five study participants got three or more meals per day. One hundred and fifty nine study participants, or 38.6% of the total, were food insecure according to the household food insecurity access scale. A total of 182 (44%) TB patients reported their habit of regular drinking of cheka while nearly one third of the study subjects (33.1%) reported to get additional food outside home (Table 2).

**Table 2:** Dietary and lifestyle characteristics of adult TB patients receiving treatment in public health facilities of conflict affected zones of Southern Ethiopia, 2023 (*n* = 414).

Variable	Characteristics	Frequency(*n* = 414)	Percent(%)
Individual Dietary Diversity Score	Poor	228	55.1
	Good	186	44.9
Meal frequency	>=3	329	79.5
	<3	85	20.5
Feeding pattern	Home	277	66.9
	Outside home	137	33.1
Regular cheka drinking	No	232	56
	Yes	182	44
Household food security	Secure	253	61.4
	Insecure	159	38.6
Drinking alcohol	No	200	48.3
	Yes	214	51.7
Smoking cigarettes	No	295	71.3
	Yes	119	28.7
Regular chat chewing	No	308	74.4
	Yes	106	25.6

a = one missing value.

**Table 3:** Health history of adult TB patients receiving treatment in public health facilities of conflict affected zones of Southern Ethiopia, 2023 (*n* = 414).

Variable	Characteristics	Frequency(*n* = 414)	Percent(%)
TB Type	Extra-pulmonary	188	45.4
	Pulmonary	226	54.6
Duration on treatment	<=2 months/intensive	94	22.7
	>2months/continuation	320	77.3
Treatment adherence a	Adherent	371	89.6
	Non-adherent	42	10.9

Two hundred and fourteen (51.7%) of the study participants reported drinking alcohol, while one hundred and nineteen (28.7%) respondents were smoking cigarettes during treatment follow-up. One hundred and six (25.6%) of study participants also reported using chat during treatment follow-up.

### Health history of the study participants

Two hundred and twenty six, (54.6%) of study subjects were diagnosed with pulmonary tuberculosis and received treatment for it, whereas one hundred and eighteen (45.4%) received treatment for extra-pulmonary tuberculosis. Regarding the length of treatment, three hundred and twenty (73.7%) got anti-TB medicine for more than two months, while forty two patients (10.9%) said they had skipped taking the drug after beginning treatment (Table 3).

### Magnitude of undernutrition

**Figure 1:**
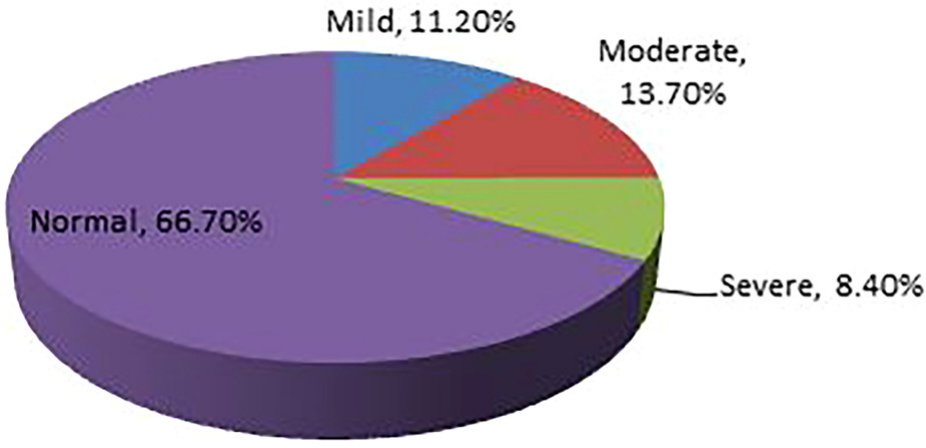
Prevalence of undernutrition among adult TB patients receiving treatment in health facilities of conflict affected zones of Southern Ethiopia, 2023.

The prevalence of undernutrition among adult tuberculosis patients in Southern Ethiopia's conflict-affected zones in 2023 was found to be 33.3% (*n* = 138) [95% CI (28.8%–38.1%)]. Of which, the percentages of mild, moderate, and severe undernutrition were 11.20%, 13.7%, and 8.4%, respectively. The BMI was 19.7 ± 2.45 (mean ± Standard Deviation) (Figure 1).

### Factors associated with undernutrition study participants

The bivariable analysis revealed that undernutrition in adult tuberculosis patients was significantly associated with the patient's sex, educational status, family size, smoking, chewing chat, drinking alcohol, frequency of meals, individual dietary diversity score, household food insecurity, and regular cheka drinking. By using multivariable logistic analysis, smoking cigarette [AOR = 2.02, 95% CI; 1.22, 3.34], chewing chat [AOR = 2.50, 95% CI; 1.59, 3.93], household food insecurity [AOR = 1.78, 95% CI; 1.19, 2.66] and regular cheka drinking [AOR = 1.82; 95% CI, 1.22–2.71] were significantly associated the outcome variable.

Respondents who drink cheka regularly were 1.82 times more likely undernourished than their counterparts who did not drink cheka regularly [AOR = 1.82; 95% CI, 1.22–2.71]. The odds of undernutrition were 2.02 times [AOR = 2.02, 95% CI; 1.22, 3.34] and 2.50 times [AOR = 2.50, 95% CI; 1.59, 3.93] higher among cigarette smokers and chat chewers than their counterparts who do not smoke cigarette and chew chat respectively. Moreover, TB patients who live in food insecure households were about 1.78 times more likely to be undernourished than those who live in food secure households [AOR = 1.78, 95% CI; 1.19, 2.66] (Table 4).

**Table 4:** Bi-variable and multivariable logistic regression analysis of factors associated with adult TB patients receiving treatment in public health facilities of conflict affected zones of Southern Ethiopia, 2023 (*n* = 414).

Variable	Category	Undernourished	Normal	COR (95% CI)	AOR (95% CI)	*p*-value
Age	≥35	58	94	1.28 (0.83–1.97)	1.18 (0.76–1.84)	0.46
	18–35 (Ref)	80	142	1.00	1.00 (Ref)	
Sex	Female	65	103	1.49 (0.96–2.30)	1.32 (0.84–2.08)	0.23
	Male	73	173	1.00 (Ref)	1.00 (Ref)	
Marital status	Married	107	221	0.89 (0.52–1.53)	0.92 (0.53–1.60)	0.77
	Single	26	48	1.00 (Ref)	1.00 (Ref)	
	Divorced	5	7	1.32 (0.38–4.60)	1.25 (0.35–4.45)	0.73
Education	≤Primary	76	126	1.46 (0.96–2.22)	1.22 (0.79–1.89)	0.37
	≥Secondary	62	150	1.00 (Ref)	1.00 (Ref)	
Occupation	Civil Servant	19	87	0.72 (0.44–1.18)	0.75 (0.45–1.24)	0.26
	Daily Laborer	61	57	1.42 (0.87–2.32)	1.51 (0.91–2.51)	0.11
	Unemployed	33	71	0.86 (0.51–1.46)	0.89 (0.52–1.53)	0.67
	Merchant	25	61	0.75 (0.41–1.37)	0.80 (0.43–1.48)	0.48
Dietary Diversity	Poor	66	163	0.89 (0.54–1.25)	0.85 (0.55–1.31)	0.45
	Good	72	113	1.00 (Ref)	1.00 (Ref)	
Meal Frequency	<3 meals/day	61	24	1.25 (0.72–2.18)	1.18 (0.67–2.09)	0.57
	≥3 meals	77	252	1.00 (Ref)	1.00 (Ref)	
Feeding Pattern	Outside home	46	91	0.95 (0.61–1.49)	0.98 (0.62–1.55)	0.93
	Home	92	185	1.00 (Ref)	1.00 (Ref)	
Cheka Drinking	Yes	105	99	1.96 (1.33–2.89)	1.82 (1.22–2.71)	**0** **.** **003**
	No	33	177	1.00 (Ref)	1.00 (Ref)	
Food Security	Insecure	96	83	1.85 (1.25–2.74)	1.78 (1.19–2.66)	**0** **.** **005**
	Secure	42	191	1.00 (Ref)	1.00 (Ref)	
Alcohol Drinking	Yes	83	131	1.12 (0.74–1.70)	1.08 (0.71–1.65)	0.72
	No (Ref)	55	145	1.00 (Ref)	1.00 (Ref)	
Smoking	Yes	35	102	2.15 (1.32–3.50)	2.02 (1.22–3.34)	**0** **.** **006**
	No	103	174	1.00 (Ref)	1.00 (Ref)	
Chat Chewing	Yes	68	58	2.72 (1.75–4.23)	2.50 (1.59–3.93)	**<0** **.** **001**
	No	70	218	1.00(Ref)	1.00(Ref)	<0.001
TB Type	Extra-pulmonary	62	126	1.00	1.00	
	Pulmonary	76	150	1.02 (0.67–1.55)	1.01 (0.66–1.54)	0.96
Duration on Treatment	>2 months	126	194	1.00 (Ref)	1.00(Ref)	
	≤2 months	12	82	0.23 (0.12–0.44)	0.87 (0.44–1.72)	0.69
Treatment Adherence	Adherent	131	240	1.00 (Ref)	1.00(Ref)	
	Non-adherent	7	35	0.37 (0.16–0.87)	0.79 (0.33–1.89)	0.60

Ref, reference; Bold *p* value represents those who have significant association.

## Discussion

This study investigated the magnitude of under-nutrition and determinant factors among adult TB patients receiving anti-TB treatment at public health facilities in the conflict affected zones of south Ethiopia. According to our research, undernutrition affected one in three adult TB patients (33.3%); this suggests that the study area had a lower prevalence of undernutrition than studies carried out in Ethiopia's Oromia and Somalia regions (38.9% and 44.3%, respectively) (47, 50). Undernutrition at the time of active tuberculosis diagnosis is one of the well-recognized risk factor for the disease progression, and predictor of relapse and death (51).

According to findings from this study patients who smoke cigarette were 2.02 times likely to be undernourished than their non-smoker counterparts. Smoking weakens the immune system and harms the lungs, increasing a smoker's risk of contracting tuberculosis. It's been demonstrated that tuberculosis is associated with a weakened immune system and various faults in immune cells (52, 53). Nicotine inhibits the expression of toll-like receptors, and the cytokine and chemokine production of type 2 pneumocytes, macrophages, and lung epithelial cells which in turn alters the inborn immune responses against tuberculosis (54, 55).

Our current findings revealed that tuberculosis patients who regularly chew chat were 2.50 times likely to be undernourished than those who do not chew. The primary psychoactive component of the plant, cathinone, is an alkaloid found in fresh leaves and shoots (56). Chewing of the leaves and shoots releases that psychoactive component; It finally suppresses appetite after being extracted into saliva and actively absorbed into the bloodstream (57, 58). On the other hand, oral cancer growth is among long-term negative impacts (59). Undernutrition in those patients with oral cancer is a result of inadequate calories intake caused by both functional and mental nature (60). Through the activation of caspases, the organic extract of chat has also been indicated to cause apoptosis (61, 62). Synergically both tuberculosis and chat reduce appetite which in turn leads to undernutrition (51, 63). Chronic gastroenteritis has been more common among chat chewers which usually disrupts nutrient absorption (64).

Cheka is a fermented beverage made mostly of cereal and vegetables that is consumed in the southwest region of Ethiopia, specifically in Derashe and Konso (44). In our current findings revealed that tuberculosis patients who regularly drink cheka were 1.82 times likely to be undernourished than those who do not drink. This can be justified by the fact that cheka has low nutrient content (65). Therefore, we recommend tuberculosis patients not to rely solely on Cheka without consuming solid foods.

Household food security is one of the well-known key determinants of undernutrition, particularly for those living in income-insecure environments (66–68). Food insecurity is also one of the predisposing factors for low individual dietary diversity (69–72). In our current study, when compared to TB patients who live in food secure households, the odds of undernutrition were 1.78 times higher among those who live in food secure households. However, our study couldn't indicate that the identified low dietary diversity attributed to either household food insecurity or other socio-demographic factors. Therefore, in order to establish clear relationship among food insecurity, dietary diversity, and undernutrition, we recommend better study designs for future researchers.

Our study's strength is in its utilization of primary and secondary data to gather as much information as possible about the factors associated with undernutrition among adult tuberculosis patients receiving treatment follow-up at health facilities in conflict-affected areas. However, during interpretation of our findings one has to consider the following limitations. First off, the cross-sectional study design used in the research meant that no causal relationship could be determined. Second, because of the study design, recall bias may have had an impact on the results. In order to increase the robustness of studies on related problems, we recommend that future research should consider these limitations. In spite of this, the study made a sincere effort to address the primary factors of undernutrition.

## Conclusions

The findings of this study show that undernutrition affects one in three adult TB patients. Lifestyle factors such as smoking and chewing, and dietary factors like household food security were significantly associated with undernutrition. In order to improve the quality of life for TB patients, it is imperative that all relevant parties give priority to addressing the lifestyle and nutritional aspects that are essential to the effectiveness of TB control and prevention initiatives.

## Data Availability

The raw data supporting the conclusions of this article will be made available by the authors, without undue reservation.

## References

[1] Maleta K. Undernutrition in Malawi. Malawi Med J. (2007) 18(4):189–205. 10.4314/mmj.v18i4.10922 PMC3345626 27529011

[2] Key M. Microonutrient-related malnutrition Who is at risk ? The United Nations Decade of Action on Nutrition (March) (2024).

[3] World Health Organization (WHO). Tuberculosis Key facts Augst 2024. WHO Fact sheet (2024). Available at: https://www.who.int/news-room/fact-sheets/detail/tuberculosis (Accessed October 29, 2024).

[4] Dye C Bassili A Bierrenbach A Broekmans J Chadha V Glaziou P Measuring tuberculosis burden, trends, and the impact of control programmes. Lancet Infect Dis. (2008) 8(4):233–43. 10.1016/S1473-3099(07)70291-8 18201929

[5] Chakaya J Khan M Ntoumi F Aklillu E Fatima R Mwaba P Global tuberculosis report 2020—reflections on the global TB burden, treatment and prevention efforts. Int J Infect Dis. (2021) 113:S7–S12. 10.1016/j.ijid.2021.02.107 33716195 PMC8433257

[6] Tb M resistant, Nations U, Development S, Calmette-gu B. Key facts Multidrug-resistant TB (2023). 2022(November).

[7] Health M of. Health Health (2009). 310–31.

[8] Seyoum B Demissie M Worku A Bekele S Aseffa A. Prevalence and drug resistance patterns of mycobacterium tuberculosis among new smear positive pulmonary tuberculosis patients in Eastern Ethiopia. Tuberc Res Treat. (2014) 2014:1–7. 10.1155/2014/753492 PMC4009208 24834351

[9] Podewils LJ Holtz T Riekstina V Skripconoka V Zarovska E Kirvelaite G Impact of malnutrition on clinical presentation, clinical course, and mortality in MDR-TB patients. Epidemiol Infect. (2011) 139(1):113–20. 10.1017/S0950268810000907 20429966

[10] Zachariah R Spielmann MP Harries AD Salaniponi FML. Moderate to severe malnutrition in patients with tuberculosis is a risk factor associated with early death. Trans R Soc Trop Med Hyg. (2002) 96(3):291–4. 10.1016/S0035-9203(02)90103-3 12174782

[11] Wagnew F Alene KA Kelly M Gray D. The effect of undernutrition on sputum culture conversion and treatment outcomes among people with multidrug-resistant tuberculosis: a systematic review and meta-analysis. Int J Infect Dis. (2023) 127:93–105. 10.1016/j.ijid.2022.11.043 36481489

[12] Abe M Akbar F Matsuura B Horiike N Onji M. Defective antigen-presenting capacity of murine dendritic cells during starvation. Nutrition. (2003) 19(3):265–9. 10.1016/S0899-9007(02)00854-7 12620532

[13] Long R. Tuberculosis and malnutrition. Int J Tuberc Lung Dis. (2004) 8(3):276–7. 15139464

[14] Izudi J Engoru S Bajunirwe F. Malnutrition is a risk factor for tuberculosis disease among household contacts: a case-control study in Uganda. IJID Reg. (2024) 12(May):100409. 10.1016/j.ijregi.2024.100409 39224535 PMC11367089

[15] Daher M. World report on violence and health. J Med Liban. (2003) 51(2):59–63. 15298158

[16] Goniewicz K Burkle FM Horne S Borowska-stefańska M Wiśniewski S Khorram-manesh A. The influence of war and conflict on infectious disease: a rapid review of historical lessons we have yet to learn. Sustainability. (2021) 13(19):10783. 10.3390/su131910783

[17] Kimbrough W Saliba V Dahab M Haskew C Checchi F. The burden of tuberculosis in crisis-affected populations: a systematic review. Lancet Infect Dis. (2012) 12(12):950–65. 10.1016/S1473-3099(12)70225-6 23174381

[18] Bélair J. Ethnic federalism and conflicts in Ethiopia. Can J Afr Stud. (2016) 50(2):295–301. 10.1080/00083968.2015.1124580

[19] Asgele Siyum B. Underlying Causes of Conflict in Ethiopia: Historical, Political, and Institutional? (2022). p. 13–26

[20] NACS. Nutrition assessment, counseling, and support (NACS): a user’s guide—module 2: nutrition assessment and classification, version 2. Nutr Assessment, Couns Support. (2016) 2:1–12. Available at: https://www.fantaproject.org/sites/default/files/resources/NACS-Users-Guide-Module2-May2016.pdf

[21] Coates J Swindale A Bilinsky P. Household Food Insecurity Access Scale (HFIAS) for Measurement of Food Access: Indicator Guide. Washington, DC: Food Nutr Tech (2007). (August): Version 3.

[22] Bursac Z Gauss CH Williams DK Hosmer DW. Purposeful selection of variables in logistic regression. Source Code Biol Med. (2008) 3:1–8. 10.1186/1751-0473-3-17 19087314 PMC2633005

[23] Bendel RB Afifi AA. Comparison of stopping rules in forward “stepwise” regression. J Am Stat Assoc. (1977) 72(357):46. 10.2307/2286904

[24] Barr RG Menzies R. The effect of war on tuberculosis. Results of a tuberculin survey among displaced persons in El Salvador and a review of the literature. Tuber Lung Dis. (1994) 75(4):251–9. 10.1016/0962-8479(94)90129-5 7949070

[25] Gele AA Bjune GA. Armed conflicts have an impact on the spread of tuberculosis: the case of the Somali regional state of Ethiopia. Confl Health. (2010) 4(1):2–7. 10.1186/1752-1505-4-1 20181042 PMC2832778

[26] Coninx R. Tuberculosis in complex emergencies. Bull W H O. (2007) 85(8):637–40. 10.2471/BLT.06.037630 17768523 PMC2636383

[27] Jayasuriya NA Nanayakkara L Iddamalgoda N Derore K. Food Security and Nutrition Among the Tuberculosis Infected Patients. A Case Study Among Patients (2015). Available at: https://documents.wfp.org/stellent/groups/public/documents/ena/wfp267386.pdf (Accessed August 26, 2014).

[28] Tomita A Ramlall S Naidu T Mthembu SS Padayatchi N Burns JK. Major depression and household food insecurity among individuals with multidrug-resistant tuberculosis (MDR-TB) in South Africa. Soc Psychiatry Psychiatr Epidemiol. (2019) 54(3):387–93. 10.1007/s00127-019-01669-y 30758540 PMC6439252

[29] Abdulla JH Gebremichael B Maruta MB Yuye I Mohammed A Debella A Nearly one out of every five adult TB patients suffered from food insecurity in Grawa District, Eastern Ethiopia: a multicenter facility-based cross-sectional study. Front Public Health. (2023) 11:1177618. 10.3389/fpubh.2023.1177618 37361149 PMC10288987

[30] Moffatt RJ Owens SG. Cessation from cigarette smoking: changes in body weight, body composition, resting metabolism, and energy consumption. Metab Clin Exp. (1991) 40(5):465–70. 10.1016/0026-0495(91)90225-L 2023532

[31] Collins LC Walker J Stamford BA. Smoking multiple high- versus low-nicotine cigarettes: impact on resting energy expenditure. Metab Clin Exp. (1996) 45(8):923–6. 10.1016/S0026-0495(96)90256-5 8769345

[32] Kennedy G Ballard T. Guidelines for Measuring Household and Individual Dietary Diversity. Rome: Fao (2010). p. 1–60. www.foodsec.org

[33] Dargie B Tesfaye G Worku A. Prevalence and associated factors of undernutrition among adult tuberculosis patients in some selected public health facilities of Addis Ababa, Ethiopia: a cross-sectional study. BMC Nutr. (2016) 2(1):1–9. 10.1186/s40795-016-0046-x

[34] Koopman JJE Van Bodegom D Beenakker KGM Jukema JW Westendorp RGJ. Hypertension in developing countries. Lancet. (2012) 380(9852):1471–2. 10.1016/S0140-6736(12)61841-8 23101715

[35] Guideline: W. Nutritional Care and Support for Patients with tuberculosis. Geneva: World Health Organization (2017). 24624480

[36] Soboka M Tesfaye M Adorjan K Krahl W Tesfaye E Yitayih Y Effect of food insecurity on mental health of patients with tuberculosis in southwest Ethiopia: a prospective cohort study. BMJ Open. (2021) 11(9):1–8. 10.1136/bmjopen-2020-045434 PMC8479992 34588229

[37] OCHA Ethiopia. Ethiopia: The Worst Drought in a Generation [Humanitarian Bulletin]. Addis Ababa: United Nations Office for the Coordination of Humanitarian Affairs (2016).

[38] CSA. Central Statistics Agency Population Size by Sex, Region, Zone and Woreda (July 2023).

[39] United Nations. Drought in Numbers 2022—Restoration for Readiness and Resilience. Covention to Combact Desertif (2022). 1–51. Available online at: https://reliefweb.int/report/world/drought-numbers-2022-restoration-readiness-and-resilience (Accessed May 13, 2022).

[40] Menbere A Feye B Getahun Z. Local conflicts and ethnic relations among konso and derashe of Southern Ethiopia: case study. Open Sci Repos Anthropol. (2013):e23050403. 10.7392/openaccess.23050403

[41] Cooper RN. Living with global imbalances: a contrarian view. J Policy Model. (2006) 28:615–27. 10.1016/j.jpolmod.2006.06.007

[42] Silva B Soares J Rocha-Pereira C Mladěnka P Remião F, On Behalf Of The Oemonom Researchers. Khat, a cultural chewing drug: a toxicokinetic and toxicodynamic summary. Toxins (Basel). (2022) 14(2):1–12. 10.3390/toxins14020071 PMC8875844 35202099

[43] Nigussie K Negash A Sertsu A Mulugeta A Tamire A Kassa O Khat chewing and associated factors among public secondary school students in Harar town, Eastern Ethiopia: a multicenter cross-sectional study. Front Psychiatry. (2023) 14:1198851. 10.3389/fpsyt.2023.1198851 37720900 PMC10499630

[44] Tsegaye B Eyayu G Zerihun E Hailu T Girma E Agedew E Proximal composition of indigenous alcoholic beverage Cheka in Konso, Southwestern, Ethiopia. J Food Process Technol. (2020) 11(8):840. 10.35248/2157-7110.20.11.840

[45] Mekonnen HS Azagew AW. Non-adherence to anti-tuberculosis treatment, reasons and associated factors among TB patients attending at Gondar town health centers, Northwest Ethiopia. BMC Res Notes. (2018) 11(1):1–8. 10.1186/s13104-018-3789-4 30285907 PMC6167840

[46] Zegeye A Dessie G Wagnew F Gebrie A Islam SMS Tesfaye B Prevalence and determinants of anti-tuberculosis treatment non-adherence in Ethiopia: a systematic review and meta-analysis. PLoS One. (2019) 14(1):1–15. 10.1371/journal.pone.0210422 PMC6328265 30629684

[47] Muse AI Ibrahim AM. Undernutrition and associated factors among adult tuberculosis patients in Jigjiga public health facilities, Somali region, East, Ethiopia. Res Rep Trop Med. (2021) 21:123–33. 10.2147/RRTM.S311476 PMC8216752 34168515

[48] Endalkachew K Ferede YM Derso T Kebede A. Prevalence and associated factors of undernutrition among adult TB patients attending Amhara national regional state hospitals, Northwest Ethiopia. Journal of Clinical Tuberculosis and Other Mycobacterial Diseases. (2022) 26:100291. 10.1016/j.jctube.2021.100291 35028435 PMC8715103

[49] Geberemeskel T Woldeyohannes D Demisie M Demisie M. Undernutrition and associated factors among adult tberculosis patients in Hossana town public health facilities, Southern Ethiopia. J Trop Dis. (2018) 06(01):123–33. 10.4172/2329-891X.1000253

[50] Tesfaye Anbese A Egeta G Mesfin F Arega Sadore A. Determinants of undernutrition among adult tuberculosis patients receiving treatment in public health institutions in Shashemane Town, Southern Ethiopia. J Nutr Metab. (2021) 2021:4218023. 10.1155/2021/4218023 34367692 PMC8339347

[51] WHO. Guideline: Nutritional Care and Support for Patients with tuberculosis. Geneva: World Heal Organ (2013). p. 1–65. Available online at: https://www.ncbi.nlm.nih.gov/books/NBK189867/24624480

[52] Davies PDO Yew WW Ganguly D Davidow AL Reichman LB Dheda K Smoking and tuberculosis: the epidemiological association and immunopathogenesis. Trans R Soc Trop Med Hyg. (2006) 100(4):291–8. 10.1016/j.trstmh.2005.06.034 16325875

[53] Altet MN Alcaide J Plans P Taberner JL Saltó E Folguera L Passive smoking and risk of pulmonary tuberculosis in children immediately following infection. A case-control study. Tuber Lung Dis. (1996) 77(6):537–44. 10.1016/S0962-8479(96)90052-0 9039447

[54] O’Leary SM Coleman MM Chew WM Morrow C McLaughlin AM Gleeson LE Cigarette smoking impairs human pulmonary immunity to mycobacterium tuberculosis. Am J Respir Crit Care Med. (2014) 190(12):1430–6. 10.1164/rccm.201407-1385OC 25390734

[55] Valdez-Miramontes CE Trejo Martínez LA Torres-Juárez F Rodríguez Carlos A Marin-Luévano SP de Haro-Acosta JP Nicotine modulates molecules of the innate immune response in epithelial cells and macrophages during infection with M. tuberculosis. Clin Exp Immunol. (2020) 199(2):230–43. 10.1111/cei.13388 31631328 PMC6954679

[56] Kalix P. Cathinone, a natural amphetamine. Pharmacol Toxicol. (1992) 70(2):77–86. 10.1111/j.1600-0773.1992.tb00434.x 1508843

[57] Kalyanasundar B Perez CI Arroyo B Moreno MG Gutierrez R. The appetite suppressant D-norpseudoephedrine (cathine) acts via D1/D2-like dopamine receptors in the nucleus accumbens shell. Front Neurosci. (2020) 14(October):1–16. 10.3389/fnins.2020.572328 33177980 PMC7596745

[58] Al-Habori M. The potential adverse effects of habitual use of Catha edulis (khat). Expert Opin Drug Saf. (2005) 4(6):1145–54. 10.1517/14740338.4.6.1145 16255671

[59] Al-Motarreb A Baker K Broadley KJ. Khat: pharmacological and medical aspects and its social use in Yemen. Phytother Res. (2002) 16(5):403–13. 10.1002/ptr.1106 12203257

[60] Gellrich NC Handschel J Holtmann H Krüskemper G. Oral cancer malnutrition impacts weight and quality of life. Nutrients. (2015) 7(4):2145–60. 10.3390/nu7042145 25825828 PMC4425137

[61] Dimba E Gjertsen BT Francis GW Johannessen AC Vintermyr OK. Catha edulis (Khat) induces cell death by apoptosis in leukemia cell lines. Ann N Y Acad Sci. (2003) 1010:384–8. 10.1196/annals.1299.070 15033757

[62] Dimba EAO Gjertsen BT Bredholt T Fossan KO Costea DE Francis GW Khat (Catha edulis)-induced apoptosis is inhibited by antagonists of caspase-1 and -8 in human leukaemia cells. Br J Cancer. (2004) 91(9):1726–34. 10.1038/sj.bjc.6602197 15477863 PMC2409956

[63] Murray CDR Le Roux CW Emmanuel A V Halket JM Przyborowska AM Kamm MA The effect of Khat (Catha edulis) as an appetite suppressant is independent of ghrelin and PYY secretion. Appetite. (2008) 51(3):747–50. 10.1016/j.appet.2008.06.012 18656509

[64] Nigussie T Gobena T Mossie A. Association between khat chewing and gastrointestinal disorders: a cross sectional study. Ethiop J Health Sci. (2013) 23(2):123–30. 23950628 PMC3742889

[65] Binitu Worku B Gemede HF Woldegiorgis AZ. Nutritional and alcoholic contents of cheka: a traditional fermented beverage in Southwestern Ethiopia. Food Sci Nutr. (2018) 6(8):2466–72. 10.1002/fsn3.854 30510748 PMC6261204

[66] Drammeh W Hamid NA Rohana AJ. Determinants of HH F-insecurity and its association with child malnutrition in Africa [Lit. Review].pdf. Curr Res Nutr Food Sci. (2019) 7(3):610–23. 10.12944/CRNFSJ.7.3.02

[67] Kshatri JS Satpathy P Sharma S Bhoi T Mishra SP Sahoo SS. Health research in the state of odisha, India: a decadal bibliometric analysis (2011-2020). J Family Med Prim Care. (2022) 11(7):3771–6. 10.4103/jfmpc.jfmpc_2192_21 36387708 PMC9648225

[68] Sethi V Maitra C Avula R Unisa S Bhalla S. Internal validity and reliability of experience-based household food insecurity scales in Indian settings. Agric Food Secur. (2017) 6(1):21. 10.1186/s40066-017-0099-3

[69] Nabuuma D Ekesa B Faber M Mbhenyane X. Food security and food sources linked to dietary diversity in rural smallholder farming households in central Uganda. AIMS Agric Food. (2021) 6(2):644–62. 10.3934/agrfood.2021038

[70] Elolu S Agako A Okello DM. Household food security, child dietary diversity and coping strategies among rural households. The case of Kole District in northern Uganda. Dialogues in Health. (2023) 3(August):100149. 10.1016/j.dialog.2023.100149 38515798 PMC10953863

[71] El Bilbeisi AH Al-Jawaldeh A Albelbeisi A Abuzerr S Elmadfa I Nasreddine L. Households’ food insecurity and their association with dietary intakes, nutrition-related knowledge, attitudes and practices among under-five children in gaza strip, palestine. Front Public Heal. (2022) 10(February):1–10. 10.3389/fpubh.2022.808700 PMC8913882 35284364

[72] Tariku A Gonete KA Bikes GA Alemu K Belew AK Wassie MM Household food insecurity predisposes to undiversified diet in Northwest Ethiopia: finding from the baseline survey of nutrition project, 2016. BMC Res Notes. (2019) 12(1):1–7. 10.1186/s13104-019-4083-9 30678698 PMC6346507

